# Functional deficits in chronic mechanical ankle instability

**DOI:** 10.1186/s13018-020-01847-8

**Published:** 2020-08-06

**Authors:** Markus Wenning, Dominic Gehring, Marlene Mauch, Hagen Schmal, Ramona Ritzmann, Jochen Paul

**Affiliations:** 1 Rennbahnklinik, Kriegackerstr 100, CH-4132 Muttenz, Basel, Switzerland; 2 Department of Orthopedic and Trauma Surgery, University Medical Center, Faculty of Medicine, University of Freiburg, Hugstetter Strasse 55, 79106 Freiburg, Germany; 3 Department of Sport and Sport Science, University of Freiburg, Schwarzwaldstrasse 175, 79117 Freiburg, Germany; 4 Department of Orthopaedic Surgery, Odense University Hospital, Sdr. Boulevard 29, 5000 Odense C, Denmark

**Keywords:** Mechanical ankle instability, Performance deficits, Peroneal dysfunction

## Abstract

**Background:**

The interaction of functional and mechanical deficits in chronic ankle instability remains a major issue in current research. After an index sprain, some patients develop sufficient coping strategies, while others require mechanical support. This study aimed to analyze persisting functional deficits in mechanically unstable ankles requiring operative stabilization.

**Methods:**

We retrospectively analyzed the functional testing of 43 patients suffering from chronic, unilateral mechanical ankle instability (MAI) and in which long-term conservative treatment had failed. Manual testing and arthroscopy confirmed mechanical instability. The functional testing included balance test, gait analysis, and concentric-concentric, isokinetic strength measurements and was compared between the non-affected and the MAI ankles.

**Results:**

Plantarflexion, supination, and pronation strength was significantly reduced in MAI ankles. A sub-analysis of the strength measurement revealed that in non-MAI ankles, the peak pronation torque was reached earlier during pronation (maximum peak torque angle at 20° vs. 14° of supination, *p* < 0.001). Furthermore, active range of motion was reduced in dorsiflexion and supination. In balance testing, patients exhibited a significant increased perimeter for the injured ankle (*p* < 0.02). During gait analysis, we observed an increased external rotation in MAI (8.7 vs. 6.8°, p<0.02).

**Conclusions:**

This study assesses functional deficits existent in a well-defined population of patients suffering from chronic MAI. Impairments of postural sway, gait asymmetries, and asymmetric isokinetic strength can be observed despite long-term functional treatment. The finding that pronation strength is particularly reduced with the foot in a close-to-accident position indicates potential muscular dysfunction in MAI. Possibly, these deficits alongside the underlying mechanical instability characterize patients requiring mechanical stabilization.

## Introduction

Perceived or subjective instability with feelings of giving way, pain, and recurrent sprains defines the clinical presentation of chronic ankle instability (CAI). This widespread phenomenon can be divided into the two etiologies of functional (FAI) and mechanical instability (MAI) [1]. The choice of the appropriate therapeutic approach for CAI patients requires the distinction between these two etiologies: functional deficits may best be addressed by functional, conservative therapy, since deficits in the sensorimotor system and postural control respond well to focused exercise regimes [2, 3]. Contrarily, MAI can only be treated mechanically using additional stabilization such as ankle taping, orthoses, or lateral ligament repair [4–6].

Concerning sensorimotor deficits, it has been described that postural control and strength are altered in FAI subjects [3, 7]. Impaired postural control has been one of the few factors which is consistently associated with functional impairment in CAI [3, 8, 9]. Furthermore, a recent systematic review found that impairments of peroneal reaction time and pronation strength strongly contribute to perceived ankle instability in a chronic population [10]. Also, strength deficits resulting from ankle injuries have been described especially in plantarflexion and pronation strength, while dorsiflexion and supination strength seems to remain rather unaffected [11, 12]. It is thought that spinal and cortical pathways may lead to an inhibition of neuromuscular activity and thus contribute to these persisting deficiencies in CAI patients [13].

Furthermore, it has been shown that CAI patients display alterations during gait as for instance a laterally deviated pressure distribution, an increased inversion angle, and a decreased foot clearance [14–16]. Whether or not these alterations during gait are caused by deficits in sensorimotor control, strength imbalance or other factors remains unclear [17–19]. However, CAI patients seem to benefit from locomotor training by improving mediolateral pressure shift during gait [18]. Furthermore, strengthening and increasing preactivation of the peroneus longus muscle have been shown to reduce ankle inversion during gait [14, 18]. On the other hand, also mechanical stabilization like ankle orthoses, taping, or operative stabilization improve gait performance by reducing maximal ankle inversion [20, 21], which can be attributed to the mechanical insufficiency which is destabilizing the joint during the ankle sprain mechanism itself [4]. Furthermore, the finding that functional performance also improves after mechanical stabilization like lateral ligament reconstruction or wearing an orthosis underlines the fact that the two etiologies are intertwined [20, 22], which of the observed alterations can be attributed to mechanical and/or functional insufficiencies is the focus of ongoing research.

Current literature suggests that a certain degree of mechanical instability cannot be compensated by functional training but may instead require mechanical stabilization [8, 23]. Whether an undetermined severity of mechanical insufficiency inevitably leads to an additional perception of instability, according to the model of Hiller et al. [24], remains unclear in current literature [4, 25].

Especially in the clinical approach to CAI patients, it is important to distinguish between those suffering from predominantly functional deficits and those patients suffering due to an insufficient mechanical stability [25, 26]. Ultimately, it may be suggested that only the latter will benefit from operative stabilization, while the others should be treated conservatively [2, 6, 27, 28]. As mentioned above, the exact definitions of CAI, FAI, and MAI have been subject to increasing debate in the last decade [29]. It is of note that many earlier studies did not differentiate the patients’ mechanical and functional insufficiencies possibly because quantifying mechanical ankle instability remains a diagnostic challenge [25]. At present, the extent to which FAI and MAI interact remains unclear [1, 10, 24, 30].

The aim of this study was to assess certain functional performance deficits in patients with unilateral, clinically apparent MAI which were scheduled for lateral ligament repair due to persisting symptoms despite previous long-term conservative treatment.

## Methods

### Study design

**Fig. 1:**
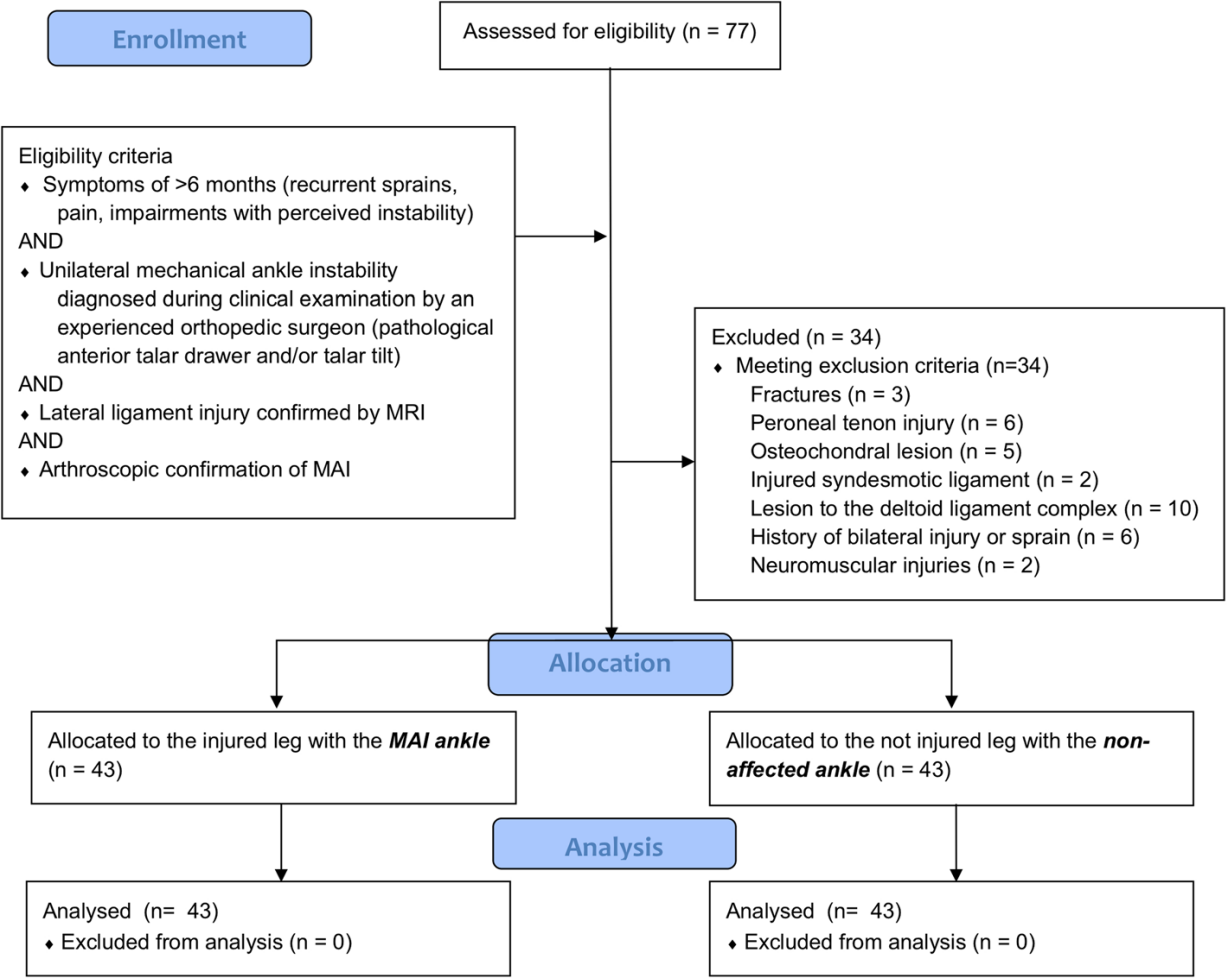
CONSORT flow chart. This figure shows the selection process of patients through the trial according to the criteria recommended in the CONSORT guidelines

We executed a single group cross-sectional retrospective analysis with a random statistical sample of 43 patients with MAI scheduled for operative lateral ligament repair which met the eligibility criteria (Fig. 1). The experiment was approved by the local ethics committee (EKNZ 2019-00241); it was retrospectively registered at the German Registry of Clinical Trials (# DRKS00021681) and conducted in accordance with the latest version of the Declaration of Helsinki.

### Participants

We screened the records of 77 patients that were scheduled for ligament repair of the talocrural joint and synthesized 43 patients (22 female and 21 male, age of 26 ± 9.8 years height 1.73 ± 0.1 m, weight of 74.1 ± 14.8 kg) complying with inclusion criteria characteristic for MAI with reference to recent publications (Fig. 1) [26, 31]. All of these patients had performed functional testing prior to surgery to assess the extent of persisting unilateral, functional deficits. They included maximal isokinetic strength measurements [3, 7], posture [3, 8, 9], and gait control [15, 16].

Chronic ankle instability was defined according to the current recommendations with ongoing symptoms of at least 6 months including recurrent sprains reported by the patients, pain, and impairments with perceived instability during sportive activity [30]. Clinical examination including the anterior talar drawer and talar tilt test was performed by an orthopedic surgeon; procedures confirmed unilateral mechanical ankle instability [32]. The included population was further diagnosed using magnetic resonance imaging (MRI) findings of one or two lateral ligament injuries (ruptures, scar tissue) [33]. During surgery, the presence of MAI was confirmed arthroscopically according to the recommendations previously described in the literature [34].

Exclusion criteria were a history of injury other than ligamentous injuries, peroneal tendon injury, osteochondral lesions on MRI, injury to the syndesmotic, or to the deltoid ligament complex. Further exclusion criteria were bilateral history of ligamentous injury or ankle sprain, Beighton score greater than four [35], fracture of the contralateral side, or other neuromuscular injuries interfering with the measurement.

According to the established treatment algorithms, all patients had received a controlled conservative treatment by an athletic trainer or physical therapist including bilateral physical therapy, strength training, and sensorimotor training for a duration of at least 3 to 6 months prior to being scheduled for surgery [2, 26]. Thus, only patients with persisting perceived instability despite previous conservative treatment were scheduled for surgery.

In accordance with the definitions in current literature [30], this forms a very homogenous cohort of patients in which unilateral MAI is the predominant pathology.

### Functional testing

The functional testing consisted of monoarticular and multiarticular tests characterized by a progressively increasing level of complexity [3, 7–9]. Isolated isokinetic strength testing was followed by a balance test (stabilometry) for the assessment of postural control and a gait analysis [15, 16] using a treadmill with integrated pedobarography.

#### Isokinetic maximal strength

Maximal strength was assessed by an isokinetic dynamometer (Humac Norm, CSMi, Stoughton, MA, USA) at a movement speed of 30°/s for plantar- and dorsiflexion. Patients laid in a prone position and the foot was tightly fixed to the footplate with Velcro straps, while the shank and the thigh were tied to the seat with a belt as described in the literature [36]. The plantarflexion/dorsiflexion axis of the upper ankle joint (best guessed by the intermalleolar axis) coincided with the rotation axis of the lever arm. For the measurements of supination and pronation, the patient was sitting semi-recumbent with the shank fixed at the leg support and the foot fixed to the footplate with Velcro straps [36, 37]. The axis of rotation was aligned with the axis of the lower ankle joint as defined by the dynamometer instructions. Prior to strength assessments, the patients performed three trials for familiarization. Recording sessions consisted of two sets with five repetitions (concentric-concentric) at maximum effort. Furthermore, active range of motion during the isokinetic strength testing was recorded. The uninjured leg was always measured first. Average measures of peak torque, peak torque angle, i.e., the angle where peak torque was reached, and range of motion out of the five trials were used for further analyses. To further evaluate the strength during the movement, we additionally extracted the torque produced across the range of motion (ROM) in steps of 5° starting from 20° of supination until 10° of pronation, since this active ROM was achieved by all participants. According to the most widely used definitions in the literature, for the purpose of this study, the most appropriate definitions of the movement around the ankle joint were supination and pronation even though internal and external rotation as well as eversion and inversion were naturally part of the motion [38, 39].

#### Postural control

For measuring postural control, a single-legged balance test was performed in a dynamic condition [40] on a ProKin Type B line system (TecnoBody, Dalmine, Italy). The subjects stood barefoot with orthograde upright posture and kept hands on their hips and directed their head forward. They were instructed to stand as still as possible, with the free leg not touching the other leg and the knee slightly flexed at each individual’s comfortable angle. Measurements started with the unaffected leg first and two trials were performed over a period of 30 s each, separated by 1 min breaks as previously described in the literature [41]. The center of pressure (COP) displacement was recorded and averaged for the medio-lateral and the anterior-posterior direction; the 90% ellipse of the COP’s area was calculated [42]. This has previously been used a valid and reliable measure of postural control [8, 41].

#### Gait analysis

Gait analysis was performed barefoot while walking on an automated treadmill equipped with integrated pressure distribution platform since overground measurement was not feasible in the available setting (FDM-T, zebris Medical GmbH, Isny, Germany). Walking speed was adjusted to the individual’s preferred gait speed between 4 and 6 km/h as described in current literatur e[43]. Spatiotemporal parameters were assessed using the integrated pressure plate. According to the literature [18, 44], the distribution of the plantar pressure along the sagittal line of the foot was measured during the stance phase and a deviation angle between the direction of gait and the sagittal orientation of the foot was calculated. Positive values equal an external rotation of the foot during stance phase. Additionally, the loading shift from posterior to anterior in percent of the stance phase was calculated to estimate gait symmetry. Deducted from the literature [45], this was done under the assumption that unequally distributed weight bearing with an increased time resting on the posterior part of the foot could be attributed to avoidance of proper active weight bearing and plantarflexion during gait.

### Statistical analysis

For statistical comparison between the non-affected and the MAI ankle, a repeated measure analysis of variance with the factor “group” (MAI affected ankle, non-affected ankle) was calculated after checking for normal distribution using Kolmogorov-Smirnov test and sphericity with Mauchly’s test. The level of significance was set at *p* < 0.05. To correct for multiple testing and resulting cumulative type I error, Bonferroni corrections were applied in the sub-analysis of pronation strength by pronation angle with 15 measurements, resulting in a significance level at *p* < 0.003 for this analysis. Values are presented as means ± standard deviation.

The statistical analysis was performed using SPSS 24 (SPSS Inc., Chicago, USA).

## Results

### Strength measurements

**Fig. 2:**
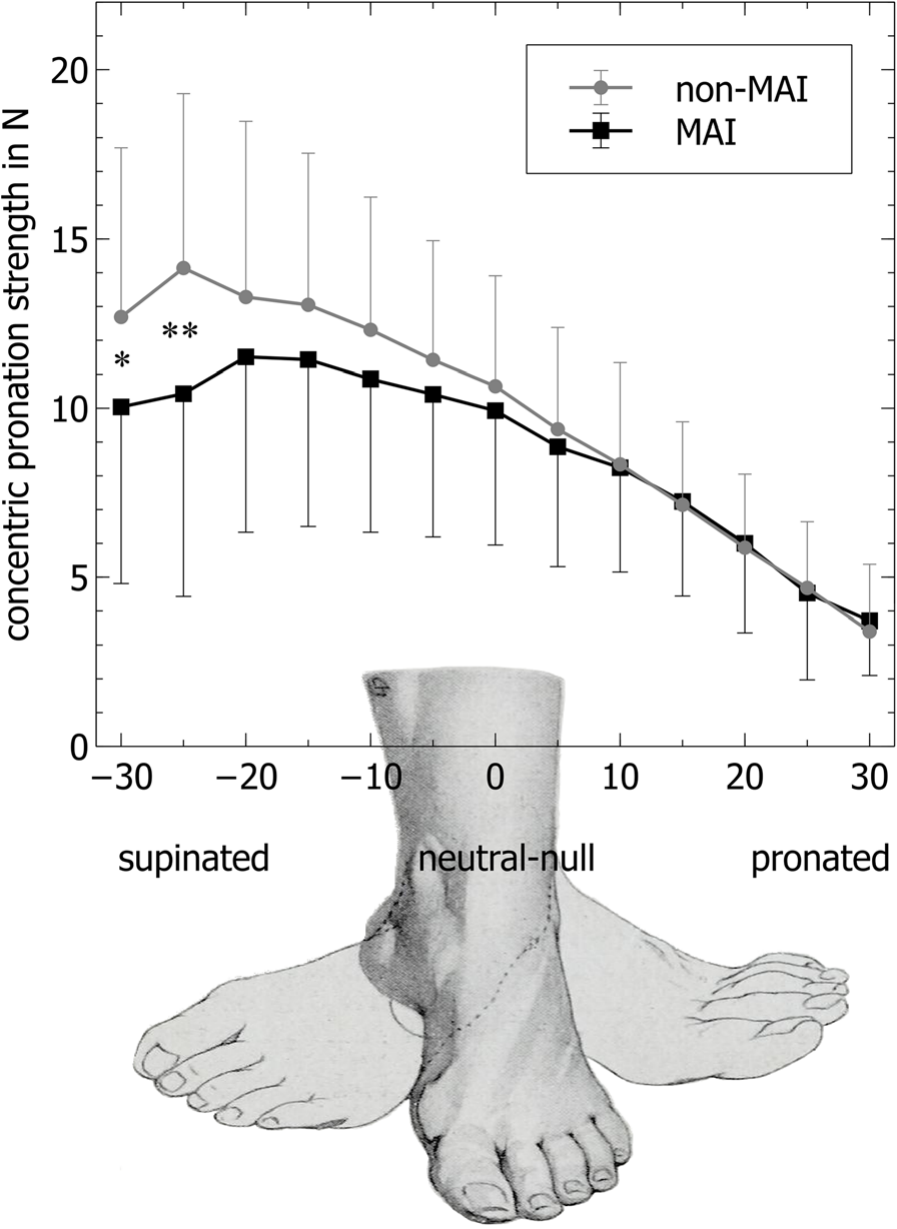
Isokinetic, concentric pronation strength according to the joint position. **p < 0.003, *p < 0.02

Statistical analysis revealed a significant reduction in maximal pronation strength (− 14%, *p* < 0.05), supination strength (− 16%, *p* < 0.05), as well as in plantarflexion strength (− 21%, *p* < 0.05), while dorsiflexion did not show a significant difference for the MAI ankle compared to the non-affected ankle joint (Table 1). The peak torque angles differed significantly between the non-affected and the MAI ankle only for the pronation strength: the MAI ankles showed a peak torque at 14° of supination, while the non-affected ankles produced the maximum pronation strength already at a 20° supination angle (Table 1). This finding is supported by the sub-analysis of pronation strength with reference to the ankle joint position: the maximum torque occurs generally during the early phase of concentric pronation in which the foot is still supinated (Fig. 2). The maximum difference in torque between the MAI and non-MAI ankle is significantly different in the early phase of pronation reflected by a difference of 3.7 Nm (MAI at 10.4 Nm and non-MAI at 14.1 Nm torque, *p* < 0.003).

**Table 1:** Isokinetic strength measurements

Isokinetic strength	Non-affected ankle (mean ± SD)	MAI ankle (mean ± SD)	ANOVA (*p*, *F*)
Maximum torque (Nm)			
Plantarflexion	**88.1 ± 28.2**	**69.8 ± 28.0**	***p*< 0.0001,*F*= 10.80**
Dorsiflexion	28.2 ± 7.6	27.9 ± 8.1	*p* = 0.68, *F* = 0.92
Supination	**24.8 ± 8.7**	**20.9 ± 9.6**	***p*< 0.001,*F*= 3.37**
Pronation	**15.9 ± 5.9**	**13.7 ± 5.6**	***p*< 0.001,*F*= 3.94**
Peak torque angle (°)			
Plantarflexion	− 5.7 ± 5.7	− 4.0 ± 6.7	*p* = 0.0603, *F* = 2.40
Dorsiflexion	12.4 ± 6.3	10.5 ± 8.1	*p* = 0.054, *F* = 1.16
Supination*	− 15.4 ± 8.5	− 15.8 ± 7.8	*p* = 0.78, *F* = 0.71
Pronation*	− **20.1 ± 7.7**	− **14.0 ± 10.7**	***p*< 0.0013,*F*= 5.41**

*Nm* Newtonmeter, *MAI* mechanical ankle instability, *ROM* range of motion

*Negative value means that peak torque for supination is achieved with the foot in pronation and vice versa

### Active range of motion

Changes in active ROM are illustrated in Table 2. The active ROM showed a significantly increased dorsiflexion by 3.6° (*p* < 0.05) and a significant decrease of 3.2° in supination in the MAI compared to the non-affected ankle joint (*p* < 0.05). The differences during active plantarflexion of 2.2° and pronation (1.1°) did not reach statistical significance (both *p* = 0.06).

**Table 2:** Active range of motion as registered by the isokinetic testing

Active ROM (°)	Non-affected ankle (mean ± SD)	MAI ankle (mean ± SD)	ANOVA (*p*, *F*)
Plantarflexion	27.2 ± 8.5	25.0 ± 8.2	*p* = 0.064, *F* = 1.16
Dorsiflexion	**17.8 ± 4.6**	**21.4 ± 4.3**	***p*< 0.0001,*F*= 3.30**
Inversion	**33.6 ± 8.6**	**30.4 ± 8.7**	***p*< 0.0401,*F*= 3.25**
Eversion	31.7 ± 7.2	30.6 ± 8.4	*p* = 0.064, *F* = 38

*MAI* mechanical ankle instability, *ROM* range of motion

### Postural control

As illustrated in Table 3, postural sway was increased in single leg stance when subjects stood on the MAI food compared to the not affected food. The MAI ankle had a 10% larger perimeter (COP-P) and a 28% augmented ellipse area of the center of pressure (COP-A) compared to the non-affected ankle.

**Table 3:** Results of the balance test examination (stabilometry)

Balance test	Non-affected ankle (mean ± SD)	MAI ankle (mean ± SD)	ANOVA (*p*, *F*)
COP-P (mm)	**1100 ± 349**	**1226 ± 446**	***p*< 0.0255,*F*= 2.61**
COP-A (mm^2^)	**515 ± 242**	**657 ± 405**	***p*< 0.0085,*F*= 4.95**

*MAI* mechanical ankle instability, *SD* standard deviation, *COP-P* path length of center of pressure, *COP-A* area (90% ellipse) of center of pressure

### Gait analysis

Spatiotemporal analysis of locomotion on the treadmill revealed that the MAI foot was significantly further externally rotated than the healthy contralateral foot by 1.9° (*p* < 0.05). The shift from rearfoot to forefoot as a measure of gait symmetry was not significantly different between the affected and the non-affected ankle (Table 4).

**Table 4:** Results of the gait analysis

Gait analysis	Non-affected ankle (mean ± SD)	MAI ankle (mean ± SD)	ANOVA (*p*, *F*)
External rotation (°)	**6.8 ± 5.1**	**8.7 ± 4.6**	***p*< 0.0155,*F*= 2.93**
Rearfoot-forefoot shift (% of stance phase)	31.8 ± 5.8	32.4 ± 9.5	*p* = 0.56, *F* = 0.26

*MAI* mechanical ankle instability, *SD* standard deviation

## Discussion

This study assessed functional deficits existent in a population of patients suffering from unilateral MAI. According to the available evidence, we chose a testing setup that reflects the different modalities of potential functional impairments: strength measurements and range of motion to assess deficits in active joint control, a test for postural sway to control for sensorimotor deficits, and gait analysis for dynamic joint control during gait [8, 19, 27, 29, 30]. While it is clear that there are numerous functional tests in the assessment of chronic ankle instability, the tests chosen result in a sound impression of potential deficits [27, 30]*.* A significant reduction in strength, an impaired postural control, and subtle changes of kinematics during locomoting were observed unilaterally on the affected side. Applying our findings to patients with persisting subjective instability despite functional treatment may help to identify those patients that will benefit from mechanical stabilization, e.g., bracing or surgery.

Comparable to our data, several studies have provided evidence that concentric pronation and supination strength are impaired in CAI [10–12]. The novel finding in this study was that the joint angle in which the maximum pronation strength can be produced is significantly different between the two ankles (MAI 14° vs. non-MAI 20° of supination). The detailed analysis according to the joint position showed that it is especially in the early phase of pronation where a significant strength deficit exists (Fig. 2). This pattern is of high clinical relevance regarding joint stabilization during gait because it will limit the ability to actively prevent excessive supination once the joint comes close to a prone-to-injury position [14, 17, 46]. The etiology behind this observation may either be related to fear of pain approaching the end-range supination, which unfortunately was not systematically recorded; it may also be a shift in torque curve due to the general decrease in ROM. The strength deficits have further been attributed to neuromuscular impairment as well as muscle atrophy [7, 47]. Also, earlier studies revealed that delayed peroneal reaction time is a characteristic of CAI patients [48], which can be improved by functional training [18]. However, since long-term functional treatment had not lead to a sufficient alleviation of symptoms in this study’s population, it may be suggested that the combination of pronounced end-range strength deficits in pronation with clinically apparent, unilateral mechanical instability results in the inability to actively prevent an ankle sprain on the previously injured side. Whether or not these unilateral impairments of end-range pronation strength are caused by the mechanical instability itself, fear of pain or posttraumatic neuromuscular dysfunction cannot be concluded. However, in our view, these patients may not be able to develop a sufficient functional performance in order to cope with their mechanical deficits. In summary, the clinical application of this finding could be that patients presenting with deficits in the end-range pronation strength and clinically apparent mechanical ankle instability will benefit from mechanical treatment, e.g., operative ligament reconstruction.

The impairments of postural sway have been widely described in the literature [10]. However, in our population, the differences in balance testing of approximal 10% were not as pronounced as in other studies [3, 7]. Furthermore, it has been shown that functional adaptations like preparatory neuromuscular activation capacitate patients to cope with mechanical insufficiencies [3, 49, 50]. Since all patients in our population had received long-term conservative treatment, it may be assumed that in these patients, the potential benefit resulting from functional training had been exhausted. Since the modalities of conservative training were not controlled for in this observational study, we are unable to conclude the reasons why conservative treatment was not successful in individual cases. From a clinical point of view, it is important to reflect upon persisting unilateral functional deficits in unilateral mechanical instability. Potentially, the observed deficit in postural control may therefore be attributed to a mechanical instability that lays beyond functional compensation. This supports the finding that the mechanical deficit itself impairs functional performance [4, 8, 46]. Again, in a clinical setting this could mean that a certain degree of mechanical instability can never be compensated and in patients with persisting perceived instability (non-copers) may require operative stabilization. Future studies need to clarify whether different degrees of severity of MAI also show different degrees of the functional deficits reported in this study. Potentially, this will also allow to estimate the risk for associated injuries following lateral ankle instability and therefore increase the therapeutic value [1, 28, 51, 52]. To achieve this, however, a reproducible manner of quantitively assessing MAI is indispensable.

Several studies have also analyzed gait in patients suffering from chronic ankle instability [21, 52]. In our analysis, we aimed to monitor whether mechanical insufficiency leads to subtle gait imbalances. While walking at a preferred speed, the distribution of weight during the stance phase showed no difference between rear- and forefoot on either leg resulting in a harmonic gait pattern. However, the foot in which mechanical instability is present was set with a slightly increased external rotation of 1.9°. This is comparable to the findings in earlier publications, where CAI had not yet been further divided into MAI vs. FAI [15, 16]. This phenomenon of a more pronounced exorotated foot may be interpreted as a subconscious preventive adaptation leading to a broader mediolateral distribution of weight due to increased support surface which will help stabilizing the ankle joint during gait [14, 18, 21]. As a limitation, it needs to be respected that kinematic adaptations take place during treadmill walking as opposed to overground [43]. However, since we compared intra-subject differences, we consider our results valid in regard to gait adaptation as a component of unilateral MAI, since the observable differences, that take place due to the treadmill condition were assumed to affect both ankles in the same way [43, 53].

Finally, to underscore these novel findings, it is promising to continue developing means for quantifying mechanical ankle instability like 3D stress-MRI [54]. Future research may then be able to establish cutoff values of severe, chronic mechanical ankle instability in which functional treatment is likely to fail and early operative stabilization should be recommended.

In addition to quantifying the individual’s mechanical insufficiency, these findings will require future confirmation and a prospective study design including FAI-patients and postoperative analysis in a clinical setting.

As a limitation, in the present retrospective analysis, we were not able to quantify the amount of perceived instability via questionnaires. However, all patients agreed to undergo operative lateral ligament repair, which makes it evident that perceived instability and subjective malfunction must have been high. Unfortunately, we were unable to retrospectively distinguish those with predominantly pathological talar tilt vs. anterior drawer in order to correlate our findings to ATFL vs. CFL rupture, which should be a focus of additional research.

## Conclusions

This retrospective analysis shows certain functional characteristics that persist in patients suffering from MAI despite functional treatment. Especially the maldistribution of peroneal strength could impede functional coping of mechanical deficits. This may support the observation that severe mechanical instability cannot be compensated and causes functional impairment itself. Thus, a certain degree of mechanical instability will eventually require operative stabilization. Future research should focus on quantifying mechanical ankle instability to verify this hypothesis.

## Data Availability

Data summarized in this study can be made available upon reasonable request.

## Abbreviations

CAI: Chronic ankle instability
MAI: Mechanical ankle instability
FAI: Functional ankle instability
ROM: Range of motion
COP: Center of pressure
